# Pulmonary Haemodynamics After Mechanical Therapy With PEA and BPA Predict Survival in Patients With CTEPH ‐ A Prospective Analysis From the National UK CURATE Registry

**DOI:** 10.1002/pul2.70419

**Published:** 2026-10-15

**Authors:** Eilish Turner‐Frick, Hakim Ghani, Paula Appenzeller, Liliana Amaral‐Almeida, Katherine Bunclark, John Cannon, Karen Sheares, Mark Toshner, Dolores Taboada, Choo Ng, John Taghavi, Steven Tsui, Nick Screaton, Alessandro Rugierro, Luigia D'Errico, Paul Bambrough, J Gerry Coghlan, David P Jenkins, Joanna Pepke‐Zaba, Stephen P Hoole

**Affiliations:** ^1^ Royal Papworth Hospital Cambridge UK; ^2^ University of Cambridge Cambridge Cambridgeshire United Kingdom

## Abstract

Chronic thromboembolic pulmonary hypertension (CTEPH) can be treated by mechanical therapy with pulmonary endarterectomy (PEA) or balloon pulmonary angioplasty (BPA). Haemodynamic treatment goal is ill‐defined. We explored post‐procedure mean pulmonary arterial pressure (mPAP), pulmonary vascular resistance (PVR), and its relationship with 5‐Year survival and exercise capacity Using 6‐min Walk Distance (6MWD). Consecutive PEA and BPA patients from the UK CURATE database, treated between 2007 and 2022 and 2015–2022, respectively, stratified by 3‐month follow‐up mPAP and PVR by right heart catheter (RHC), had 5‐year survival and 6MWD compared, adjusted for age, sex, drug treatment, and comorbidities in the PEA cohort. Those treated by PEA achieving an mPAP ≤ 25 mmHg had significantly better survival than higher mPAP groups (all pairwise comparisons *p* < 0.001), with no additional survival advantage below mPAP of 25 mmHg. Those with PVR ≤ 300 dyne.s.cm^−5^ also had better 5‐year survival (*p* < 0.001). Continuous PVR modelling showed a 17% decrease in 5‐year survival for every 100 dyne.s.cm^−5^ increase in post‐operative PVR (*p* < 0.001). And these results remained significant after proportional hazard modelling. Those treated by BPA achieving an mPAP ≤ 30 mmHg and PVR ≤ 300 had a significantly better 5‐year survival with no additional survival advantage below these values. Continuous PVR modelling showed a 48% decrease in 5‐year survival for every 100 dyne.s.cm^−5^ increase in post‐operative PVR (*p* = 0.008). In conclusion, post‐procedure haemodynamics predict 5‐year survival after mechanical therapy for CTEPH. Achieving a mPAP ≤ 25 mmHg for PEA and ≤ 30 mmHg for BPA, or a PVR ≤ 300 dyne.s.cm^−5^ for both, conferred a better prognosis.

## 1 Introduction

Chronic thromboembolic pulmonary hypertension (CTEPH) is caused by persistent obstruction of pulmonary arteries by organised thromboemboli in around 2.7% in pulmonary emboli survivors [1]. Two mechanical treatments are available for CTEPH: pulmonary endarterectomy (PEA) or when surgical risk is high or the disease is distributed distally, balloon pulmonary angioplasty (BPA).

Whilst most clinicians intuitively believe “lower is better,” there is currently no universally agreed measure of haemodynamic success for mechanical treatment of CTEPH which could offer reassurance and guide future treatment decisions [2, 3]. In patients undergoing BPA, where multiple procedures are needed, pulmonary haemodynamics could offer guidance on when to stop offering further BPA, which is pertinent in higher‐risk procedures and in health economies with limited resources.

We assessed the relationship between the final haemodynamic result following mechanical treatment of CTEPH and medium‐term survival and exercise capacity in consecutive patients undergoing PEA and BPA to establish haemodynamic predictors of treatment success.

### 1.1 Methods

Using data from the CTEPH UK Registry to Assess Treatment outcomEs (CURATE) database, consecutive patients treated at the national PEA centre at Royal Papworth Hospital, Cambridge, UK between 2007 and 2022 (PEA cohort) and 2015–2022 (BPA cohort) were prospective recorded to enable comparison of 3‐month follow‐up haemodynamic assessments from right heart catheterisation (RHC) following completion of either PEA or BPA with survival outcomes. Patients who died before 3‐month follow‐up, those lost to follow‐up, and those without 3‐month haemodynamic value were excluded. Patients who had repeat PEA or BPA after PEA were not double‐counted.

Patients were divided into three groups based on their follow‐up mean pulmonary artery pressure (mPAP) and pulmonary vascular resistance (PVR). For mPAP, patients in both cohorts were divided up into the following groups: ≤ 30, 30–40, and > 40 mmHg; in the PEA cohort, the ≤ 30 mmHg group was later further stratified into ≤ 20, 21–25, and 26–30 mmHg subgroups for more detailed comparisons of PH‐defined cut points. The haemodynamic tertiles for PVR were: ≤ 300, 300–600, and >600 dyne.s.cm^−5^. The PVR for the PEA cohort was again further stratified into ≤ 160, 161–240, 241–320, 321–400, and >400 dyne.s.cm^−5^ subgroups.

Outcome measures at 5‐years following their 3‐month follow up assessment included survival, and 6‐min walk distance (6MWD) at 3‐months.

### 1.2 Statistics

Kaplan–Meier curves were analysed by pairwise log‐rank test with Benjamini–Hochberg correction to compare PEA and BPA cohorts at each haemodynamic stratum for both survival and 6MWD. In our substantially larger PEA cohort, Cox proportional hazard models (including Schoenfeld residuals) were used to adjust for potential confounders (age, sex, comorbidities (previous splenectomy, diabetes, hypertension, dyslipidaemia, atrial fibrillation, a thyroid condition, or ischaemic heart disease), and PAH medication use post‐surgery). We also conducted restricted cubic spline modelling to assess whether the relationship between continuous post‐operative mPAP or PVR and mortality hazard was non‐linear. Differences in follow‐up 6MWD between haemodynamic groups were assessed using pairwise log‐rank testing for our categorical analysis with Benjamini–Hochberg correction and any association between continuous post‐treatment mPAP and PVR and follow‐up 6MWD was assessed using linear regression. We used ANCOVA to adjust for baseline 6MWD both in categorical and continuous analysis. In our BPA cohort, as the numbers were too small, to avoid too many confounders, we used unadjusted analysis.

## 2 Results

A Consort diagram for the patients treated by PEA and BPA is summarised in Appendix Figure 1. Median follow‐up was 5.0 years (95% CI 5.0–5.0) in the PEA cohort and 4.21 (95% CI: 3.69–4.57) in the BPA cohort. The demographic and clinical characteristics of each group are summarised in Appendix Table 1.

### 2.1 Post PEA

The 5‐year mortality for the PEA cohort was 8.2%. PEA patients with post‐operative mPAP of > 30–40 and > 40 mmHg showed a statistically significant lower survival compared to patients who achieved an mPAP of ≤ 30 mmHg (Figure 1A, *p *< 0.001, Appendix Table 2). Stratifying the mPAP ≤ 30 mmHg group further into ≤ 20 mmHg, > 20–25 mmHg and > 25–30 mmHg subgroups, demonstrated a statistically significant survival difference between those with an mPAP ≤ 25 mmHg (*n* = 975, 41 deaths, 5‐year KM survival 94.5% (95% CI 92.9–96.2%)) and mPAP > 25 mmHg (*n* = 762, 762 death, 5‐year KM survival 84.5% (95% CI 81.2–87.0%, *p* < 0.001) with an adjusted HR 2.56 (95%CI 1.73–3.78) *p* < 0.001, Figure 1A. A non‐statistically significant difference was observed between the subgroups ≤ 20 mmHg (*n* = 546, deaths 22, 5 year KM survival 94.7% (95% CI 92.6–96.9%) and > 20–25 mmHg (*n* = 429, 19 deaths, 5 year KM survival 94.3% (95% CI 91.8–96.8%), *p* = 0.77. Using a Cox proportional hazard regression with restricted cubic splines, we found there was evidence of non‐linearity in the relationship between mPAP and survival (*p* = 0.01, Appendix Figure 2). Using maximally selected log‐rank statistic, the data‐derived cut point with the greatest separation in survival was an mPAP of 27 mmHg (*p* = 3.8 × 10^−12^).

**FIGURE 1:**
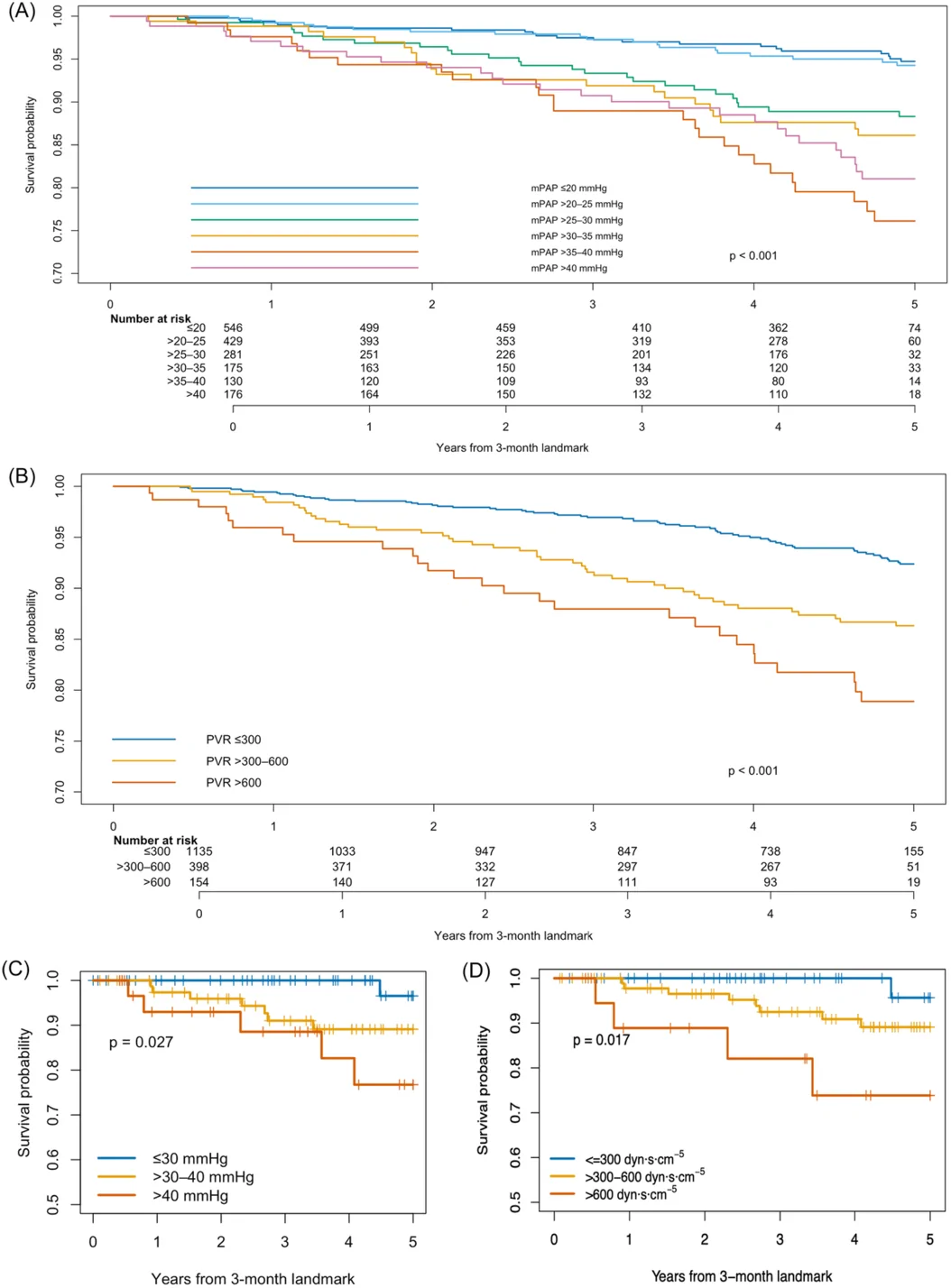
(A) Kaplan–Meier curve showing 5‐year conditional survival after pulmonary endarterectomy (PEA) surgery for mPAP; (B) Kaplan–Meier curve showing 5‐year conditional survival after PEA surgery for PVR; (C) Kaplan–Meier curve showing 5‐year conditional survival after balloon pulmonary angioplasty (BPA) for mPAP; (D) Kaplan–Meier curve showing 5‐year conditional survival after BPA for PVR.

PEA patients with a post‐operative PVR of > 300–600 dyne.s.cm^−5^ and > 600 dyne.s. cm^−^ had statistically significant lower 5‐year survival than the PVR ≤ 300 dyne.s.cm^−5^ (*p* < 0.001, Figure 1B, Appendix Table 2) with an adjusted HR 1.88 (95% CI 1.18–3.02); *p* = 0.008; and adjusted HR 2.26 (95% CI 1.16–4.40); *p* = 0.016, respectively (Figure 1B).

Pairwise log‐rank testing with Benjamin–Hochberg correction demonstrated difference between ≤ 300 dyne.s.cm^−5^ and > 300–600 dyne.s.cm^−5^ as well as > 600 dyne.s.cm^−5^ (*p* < 0.001) but not between > 300–600 and > 600 dyne.s.cm^−5^ (*p* = 0.054). Cox proportional hazards regression with restricted cubic splines found that PVR and survival also showed a non‐linear relationship (*p* = 0.004)) with a maximally selected log‐rank statistic identifying 378 dyne.s.cm^−5^ as the strongest data‐derived cut‐point with the greatest separation in survival (*p* = 3.05 × 10^−^^6^) (Appendix Figure 3).

dyne.s. cm Stratifying the post PEA PVR ≤ 400 dyne.s.cm^−5^ group into subgroups confirmed that that there was no additional survival benefit below 240 dyne.s.cm^−5^ but the other PVR subgroups <400 dyne.s. cm^−5^ had a step wise improvement in survival (*p* = 0.003; Appendix Figure 4). When PVR was analysed as a continuous variable, there was a 17% decrease in 5–year survival for every 100 dyne.s.cm^−5^ increase in post‐operative PVR (HR 1.17 (95% CI 1.11–1.23); *p* < 0.001).

The survival of post‐PEA patients with an mPAP > 25 mmHg was not influenced by PVR; both mPAP> 25 mmHg/PVR < 300 dyne.s. cm^−5^ (*n* = 268, 5‐year survival 85.1% (95% CI 80.3–90.2)) and mPAP > 25 mmHg/PVR ≥ 300 dyne.s.cm^−5^ (*n* = 466, 5‐year survival 82.9% (95% CI 79.2–86.7)) had similar poorer survivals when compared to those with mPAP ≤ 25 mmHg/PVR < 300 dyne.s.cm^−5^ (5‐year survival 94.6% (95%CI 92.9–96.4), both *p* < 0.001. However, in the mPAP >25 mmHg/PVR < 300 dyne.s.cm^−5^ group, poorer survival appeared to be driven predominantly by higher PCWP rather than higher CO when dichotomising further for these variables (5‐year survival PCWP ≤ 14 mmHg: 90.8% (95% CI 85.5%–96.5%) vs. > 14 mmHg: 79.7% (95% CI 71.9–88.4%), *p* = 0.05 and CO ≤ 5.5 L/min: 84.9% (95% CI 78.4–92.1%) vs. CO > 5.5 L/min: 85.7% (78.9–93.1%), *p* = 0.82).

### 2.2 Post BPA

The 5‐year mortality for the BPA cohort was 6.7%. Those with an mPAP ≤ 30 mmHg showed increased 5‐year survival compared to the higher mPAP group > 40 mmHg group (*p* = 0.014, unadjusted HR 11.58 (95% CI 1.35–99.14), *p* = 0.025), and a trend in improved survival compared to > 30–40 mmHg, (*p* = 0.103, unadjusted HR 5.67 (95% CI 0.70–46.10), *p* = 0.105) (Figure 1C, Appendix Table 3). There was no statistically significant difference in 5‐year survival between ≤ 30 mmHg and the > 30–40 mmHg groups.

BPA patients with a post‐operative PVR ≤ 300 dyne.s.cm^−5^ (had a significantly better survival than those with PVR > 600 dyne.s.cm^−5^ (*p* = 0.009; unadjusted HR 2.97 (95% CI 1.15–116.10), *p* = 0.022, Figure 1D, Appendix Table 3). There were no other significant differences between 5‐year survival between other PVR comparisons. We did not further subdivide the BPA haemodynamic categorisation due to small numbers in this cohort. When PVR was analysed as a continuous variable, there was a 42% decrease in 5‐year survival for every 100 dyne.s.cm^−5^ increase in post‐BPA PVR (HR 1.42 (95% CI 1.16–1.74); *p* < 0.001).

## 3 Discussion

In this study, 5‐year survival rates were found to correlate with post‐procedure haemodynamics after PEA and BPA and demonstrated a haemodynamic threshold for this improvement of < 25 mmHg for PEA and < 30 mmHg for BPA with a PVR threshold ≤ 300 dyne.s.cm^−5^ for improvement in both cohorts. Final achieved haemodynamics in the PEA cohort also correlated with improved 6MWD.

These findings are consistent with a previous study [4], which also demonstrated a prognostic threshold of < 30 mmHg for BPA patients. In the current study, the mPAP threshold after PEA of < 25 mmHg for prognostic benefit was lower than our previously reported adjusted prognostic threshold of 38 mmHg [5]. In this study, the most discriminative cut points for survival in the PEA cohort were mPAP 27 mmHg and PVR 378 dyne.s.cm^−5^. Those with higher mPAP and discordant lower PVR still had a poor prognosis, with a high PCWP rather than high CO driving the poorer prognosis in this group, perhaps reflecting the importance of occult LV diastolic dysfunction, that is, uncovered after mechanical CTEPH treatment.

Whereas PEA surgery is undertaken as a single definitive intervention, BPA is performed serially with the opportunity to be guided by the prevailing haemodynamics. As such, a haemodynamic threshold for prognostic benefit is of particular value for BPA. A technically challenging BPA, which carries increased risk, may be deferred in a patient with an mPAP < 30 mmHg and symptom control, as the 5‐year survival is good and does not appear to be further improved by achieving a lower mPAP or PVR. However, a prospective treat‐to‐target survival comparison is needed to confirm this view.

Right ventricular failure is predominantly the mechanism of death in patients with untreated CTEPH, and it has been shown that mechanical treatments for CTEPH reduce RV volume and improve RV function (1A). We now hypothesise that, unlike PAH, once a mPAP threshold < 25 mmHg is reached, RV remodelling restores RV size and function to a safe level in mechanically treated CTEPH patients, resulting in a plateaued improvement in long‐term survival, irrespective of further haemodynamic improvement; survival is then determined by non‐CTEPH factors and general cardiovascular risk (2A).

Our results in the PEA group in particular, demonstrate that post‐operative mPAP ≤ 25 mmHg and PVR < 300 dyne.s.cm^−5^ were also associated with an increase in follow‐up 6MWD. We have previously published that PVR predicts 6MWD in PEA‐treated patients [5]. Our BPA cohort may have been underpowered to demonstrate a similar relationship between improved 6MWD and haemodynamics, as other studies have shown a similar relationship after BPA (3A).

## 4 Study Limitations

The sample size for our BPA cohort was modest, and due to the nature of registry datasets, several patients had incomplete haemodynamic or functional data. Despite this, we believe our PEA findings are robust, and the PEA and BPA survival data align. There were very few events (12 deaths) in the BPA group, which likely explains the challenges in determining a strong PVR relationship with mortality. The BPA data remain unadjusted and should be viewed as hypothesis‐generating that needs to be confirmed with larger datasets. Furthermore, registry data can only attribute an association with achieved haemodynamic target and outcome. We have not been able to account for the potential confounding effect of drug treatment changes during follow‐up on the relationship between haemodynamics and survival. However, as drug treatment changes are invariably driven by the patient's haemodynamic status, accounting for these changes should only strengthen rather than weaken the relationship we observed. The change in use of drug therapy may have particularly weakened associations in the smaller BPA cohort. Finally, our dataset is from a single established site providing a national service for PEA and BPA treatment but may not reflect data from other national centres, due to procedural and technical differences in approach that may be different site‐to‐site, and this may limit the generalisability of our findings.

## 5 Conclusion

This study demonstrated a favourable prognosis for patients with CTEPH undergoing mechanical treatment when post‐procedure mPAP values achieved are below 25 mmHg for PEA and below 30 mmHg for BPA and PVR is < 300 dyne.s.cm^−5^ for both. These data are particularly useful in counselling future patients, deciding treatment escalation, and in particular, justifying further BPA to achieve better haemodynamics in an asymptomatic patient.

## Author Contributions

Eilish Turner‐Frick contributed to study design, data analysis and interpretation, and drafting of the manuscript. Dr Stephen Hoole and Dr Hakim Ghani contributed to the study design and conception and supervised the study as well as manuscript drafting. All other authors critically revised the manuscript and approved the final version. All authors are guarantors of the manuscript.

## Funding

The authors have nothing to report.

## Ethics Statement

This study involves human participants. This was a secondary analysis of a registry and constituted a service evaluation, and thus did not require ethical approval from an ethics committee and active consent from patients. We have used the National Data Opt‐outs service through MESH and have excluded patients who have opted out for their data to be used.

## Conflicts of Interest

Royal Papworth Hospital and University of Cambridge.

## Supporting information


**Figure S1:** Consort diagram depicting patient flow within each cohort.

**Figure S2:** Cox proportional hazards regression model with restricted cubic splines demonstrating non‐linearity between mPAP and 5‐year survival.

**Figure S3:** Cox proportional hazards regression model with restricted cubic splines demonstrating non‐linearity between PVR and 5‐year survival.

**Figure S4:** Kaplan‐Meier curve showing 5‐year conditional survival after PEA surgery for PVR stratified for lower and narrower subgroups.

**Figure S5:** (A) Box chart showing differences in follow‐up 6MWD for mPAP in the PEA cohort. (B) Box chart showing differences in follow‐up 6MWD for mPAP in the BPA cohort.

**Table S1:** Baseline demographic and clinical characteristics for Pulmonary Endarterectomy (PEA) and Balloon Pulmonary Angioplasty (BPA) patients.
**Table S2:** Summary of the 5‐year KM survival and 6MWD for the PEA cohort stratified by post‐operative haemodynamic data.
**Table S3** Summary of the 5‐year KM survival and 6MWD for the BPA cohort stratified by post‐operative haemodynamic data.

Supporting File
