# Supplementary material for: Pulmonary Haemodynamics After Mechanical Therapy With PEA and BPA Predict Survival in Patients With CTEPH ‐ A Prospective Analysis From the National UK CURATE Registry
Source: Pulm Circ. 2026 Oct 15;16(4):e70419. doi: 10.1002/pul2.70419 (PMC13669816; doi:10.1002/pul2.70419)
Supplement: Supplementary file 1 [file PUL2-16-e70419-s005.docx]

**Appendix Tables**

**Appendix Table 1:** Baseline demographic and clinical characteristics for Pulmonary Endarterectomy (PEA) and Balloon Pulmonary Angioplasty (BPA) patients.

|  | **PEA** | **BPA** | **p-value** |
| --- | --- | --- | --- |
| **Demographics** |  |  |  |
| Age (years) | 57.9± 15.1 (1742) | 63.3± 12.8. (1742) | <0.001 |
| BMI (kg/m²) | 29.9 ± 6.8 (1742) | 26.7 ± 6.1 (1742) | <0.001 |
| Male Sex | 954/1739 (54.9%) | 97/178 (54.5%) | 0.919 |
| Vasodilator therapy | 625/1416 (44.1%) (2015-2022) | 170/178 (95.5%) | <0.001 |
| Hypertension | 460/1696 (27.1%) | 45/175 (25.7%) | 0.689 |
| Dyslipidaemia | 141/1460 (9.7%) | 8/175 (4.6%) | 0.015 |
| Thyroid disorder | 137/1695 (8.1%) | 23/175 (13.1%) | 0.025 |
| Splenectomy | 5/1702 (3.1%) | 19 (10.9%) | <0.001 |
| Diabetes | 175/1695 (10.3%) |  |  |
| Atrial Fibrillation | 131/1696 (7.7%) |  |  |
| **Functional status** |  |  |  |
| 6MWD (m) | 306.5 ± 144.3 | 349.9 ± 128.0 | <0.001 |
| WHO Functional Class |  |  | <0.001 |
| I | 2 | 0 |  |
| II | 260 | 50 (28.4%) |  |
| III | 1190 | 125 (71%) |  |
| IV | 124 (1576) | 1 (0.6) (176) |  |
| **Haemodynamics (preop)** |  |  |  |
| PVR (dyne.s.cm⁻⁵) | 594 ± 355 (1726) | 562 ± 244 (174) | 0.119 |
| mPAP (mmHg) | 45.8± 15.8 (1727) | 41.1 ± 9.9 (176) | <0.001 |
| PCWP (mmHg) | 11.0 ± 4.4 (1584) | 9.5 ± 3.2 (176) | <0.001 |
| CO (thermodilution) | 4.9± 1.9 (1726) | 4.8 ± 1.1 (172) | 0.157 |
| RAP (mmHg) | 11.3 ± 5.6 (1584) | 6.8 ± 3.7 (172) | <0.001 |

Data are presented mean ± SD or n (%).

Different denominators based on data availability n in brackets.

BMI – body mass index; 6MWD – 6-minute walk distance; WHO – World Health Organisation; PVR – pulmonary vascular resistance; mPAP – mean pulmonary artery pressure; PCWP – pulmonary capillary wedge pressure; CO – cardiac output; RAP – right atrial pressure.

**Appendix Table 2:** Summary of the 5-year KM survival and 6MWD for the PEA cohort stratified by post-operative haemodynamic data

| **Haemodynamic stratum** | **n** | **Deaths** | **5-year-KM survival % ( 95% CI)** | **Unadjusted HR (95% CI)** | **P-value** | **Follow-up 6MWD median (IQR)** | **6MWD (n)** |
| --- | --- | --- | --- | --- | --- | --- | --- |
| **mPAP ≤30mmHg** | 1256 | 67 | 93.1 (91.5-94.8) | Reference |  | 396 (314-466) | 1104 |
| **mPAP >30-40mmHg** | 305 | 46 | 82.0 (77.4-86.9) | 2.78 (1.91-4.05) | <0.001 | 326 (232-398) | 259 |
| **mPAP >40mmHg** | 176 | 27 | 81.0 (74.7-87.9) | 2.90 (1.85-4.53) | <0.001 | 290 (210-366) | 139 |
| **PVR ≤300 dyne.s.cm⁻⁵** | 1135 | 65 | 92.4 (90.6-94.2) | Reference |  | 400 (322-470) | 991 |
| **PVR >300-600 dyne.s.cm⁻⁵** | 398 | 46 | 86.3 (82.7-90.1) | 1.98 (1.36-2.89) | <0.001 | 320 (256-400) | 338 |
| **PVR >600 dyne.s.cm⁻⁵** | 154 | 27 | 78.9 (72.0-86.4) | 3.17 (2.03-4.97) | <0.001 | 300 (210-363) | 129 |

**Appendix Table 3:** Summary of the 5-year KM survival and 6MWD for the BPA cohort stratified by post-operative haemodynamic data

| **Haemodynamic stratum** | **n** | **Deaths** | **5-year KM survival %**  **(95% CI)** | **Unadjusted HR**  **(95% CI)** | **P-value** | **Follow-up 6MWD, median**  **(IQR)** | **6MWD (n)** |
| --- | --- | --- | --- | --- | --- | --- | --- |
| **mPAP ≤30mmHg** | 66 | 1 | 96.6 (90.1–100) | Reference |  | 434 (341-509) | 58 |
| **mPAP >30-40mmHg** | 80 | 7 | 89.1 (81.7–97.2) | 5.67 (0.70–46.10) | 0.105 | 390 (284–477) | 67 |
| **mPAP >40mmHg** | 32 | 5 | 76.7 (60.2–97.8) | 11.58 (1.35–99.14) | 0.025 | 418 (322–463) | 28 |
| **PVR ≤300 dyne.s.cm⁻⁵** | 59 | 1 | 95.7 (87.7–100) | Reference |  | 441 (364–510) | 51 |
| **PVR >300-600 dyne.s.cm⁻⁵** | 96 | 8 | 89.1 (82.1–96.7) | 4.41 (0.55–35.30) | 0.162 | 392 (301–474) | 82 |
| **PVR >600 dyne.s.cm⁻⁵** | 20 | 4 | 73.8 (54.3–100) | 12.97 (1.45–116.10) | 0.022 | 344 (280–417) | 17 |

**6MWD in the PEA and BPA groups**

The mPAP ≤30 mmHg group performed significantly better 6MWD compared to those >30 mmHg (p-values <0.0001) and there was also statistically significant difference in the 3-month follow-up 6MWD between the >30-40 mmHg and >40 mmHg groups (p=0.032), Appendix Figure 5A. Subdividing the ≤30 mmHg group further we also saw a statistically significant difference in 6MWD walking distance between the ≤25 mmHg subgroup and those >25 mmHg. After adjusting for baseline 6MWD, follow-up 6MWD continued to differ significantly across post-PEA mPAP categories (p<0.001).

Both >300-600 and >600 dyne.s.cm⁻⁵ PVR groups, were associated with a significantly lower 6MWD compared to the ≤300 dyne.s.cm⁻⁵ group (p<0.001). In addition, 6MWD was significantly lower in >600 dyne.s.cm⁻⁵ compared to >300-600 dyne.s.cm⁻⁵ (p=0.012). After adjustment for baseline 6MWD, follow-up 6MWD remained significantly different across postoperative PVR categories (p < 0.001). The same was observed when further stratifying the PEA cohort into narrower PVR subgroups.

There were no significant differences between the different PVR and mPAP groups in the BPA cohort and 6MWD (Appendix Figure 5B).

**Appendix references:**

1A: Hoole SP, Coghlan JG, Cannon JE, Taboada D, Toshner M, Sheares K, et al. Balloon pulmonary angioplasty for inoperable chronic thromboembolic pulmonary hypertension: the UK experience. Open Heart. 2020;7(1): e001144. doi: 10.1136/openhrt-2019-001144.

2A: Liley J, Bunclark K, Newnham M, Cannon J, Sheares K, Taboada D, et al. Development of an open-source tool for risk assessment in pulmonary endarterectomy. Eur Respir J. 2025;65(3):2401001. doi: 10.1183/13993003.01001-2024.

3A: Daigo K, Katsumata Y, Esaki K, Iwasawa Y, Ichihara G, Miura K, et al. Predictors of Improvement in Exercise Tolerance After Balloon Pulmonary Angioplasty for Chronic Thromboembolic Pulmonary Hypertension. J Am Heart Assoc. 2023;12(3): e8137. doi: 10.1161/JAHA.122.027395.
